# Supplementary material for: Aeolian sediment transport on Io from lava–frost interactions
Source: Nat Commun. 2022 Apr 19;13:2076. doi: 10.1038/s41467-022-29682-x (PMC9018742; doi:10.1038/s41467-022-29682-x)
Supplement: Supplementary file 1 — Supplementary Information [file 41467_2022_29682_MOESM1_ESM.pdf]

Supplementary Information

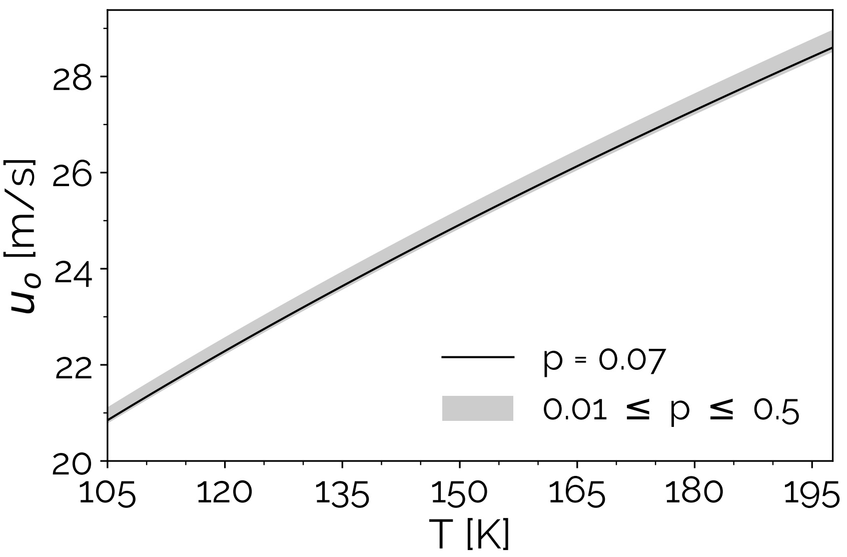

Supplementary Figure 1: **Outgassing velocities.** The outgassing velocity u_0 for SO_2 under a porous sublayer, as a function of surface temperature. The line uses a value of 0.07 for p_c, the probability for a molecule to cross the porous layer. The shaded region denotes the range for $0.01 < p_c \leq 0.5$.

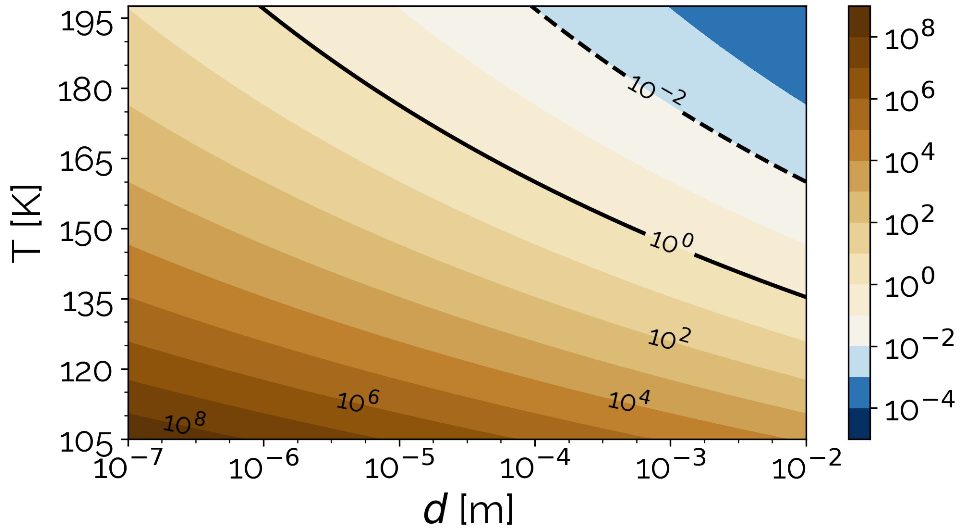

Supplementary Figure 2: **Knudsen number.** As a function of temperature and grain diameter d. The critical Knudsen number of 0.01, above which the Cunningham correction for drag is used in the saltation relations, is denoted by the dashed line. A Knudsen number of 1 is also marked for reference with a solid line.

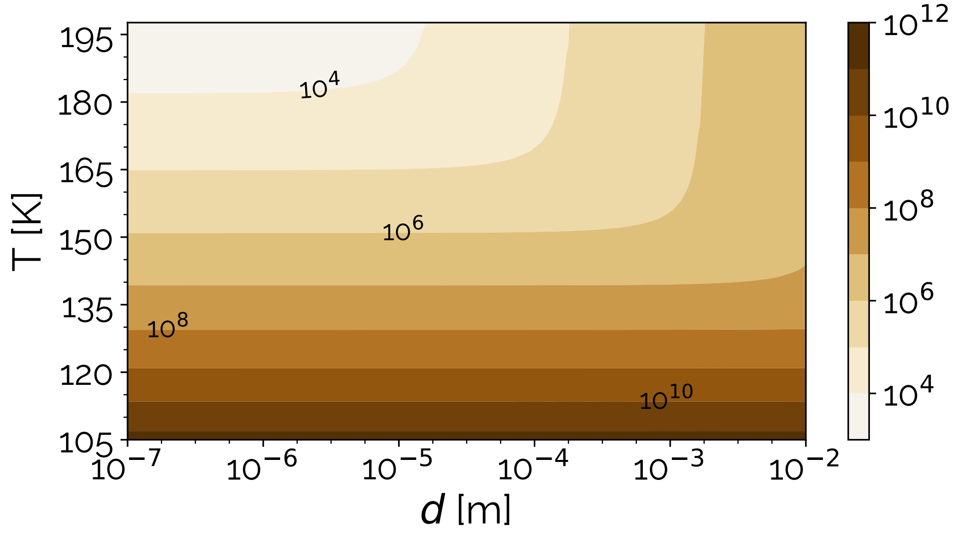

Supplementary Figure 3: **Stokes number.** As a function of temperature and grain diameter d.

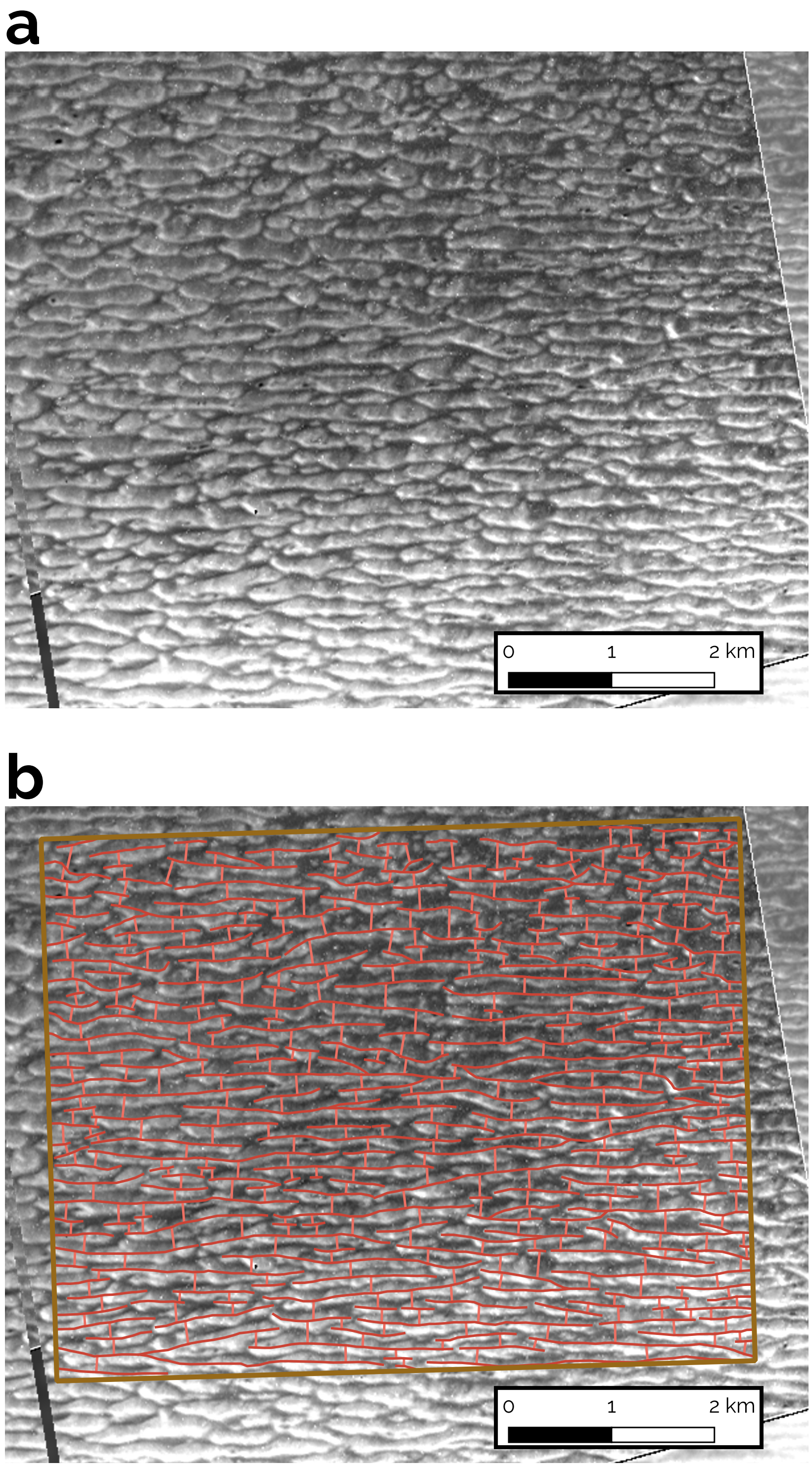

Supplementary Figure 4: **Digitization of ridges for the Prometheus Patera site. a,** Shows the original *Galileo* Solid State Imager image, unmarked. **b,** Crestlines are marked in dark red, with the spacing between each adjacent crest marked in light red. The boundary box within which crests were digitized is marked in brown. The image ID and latitude/longitude for this and other measured ridge sites are tabulated in Supplementary Table 2.

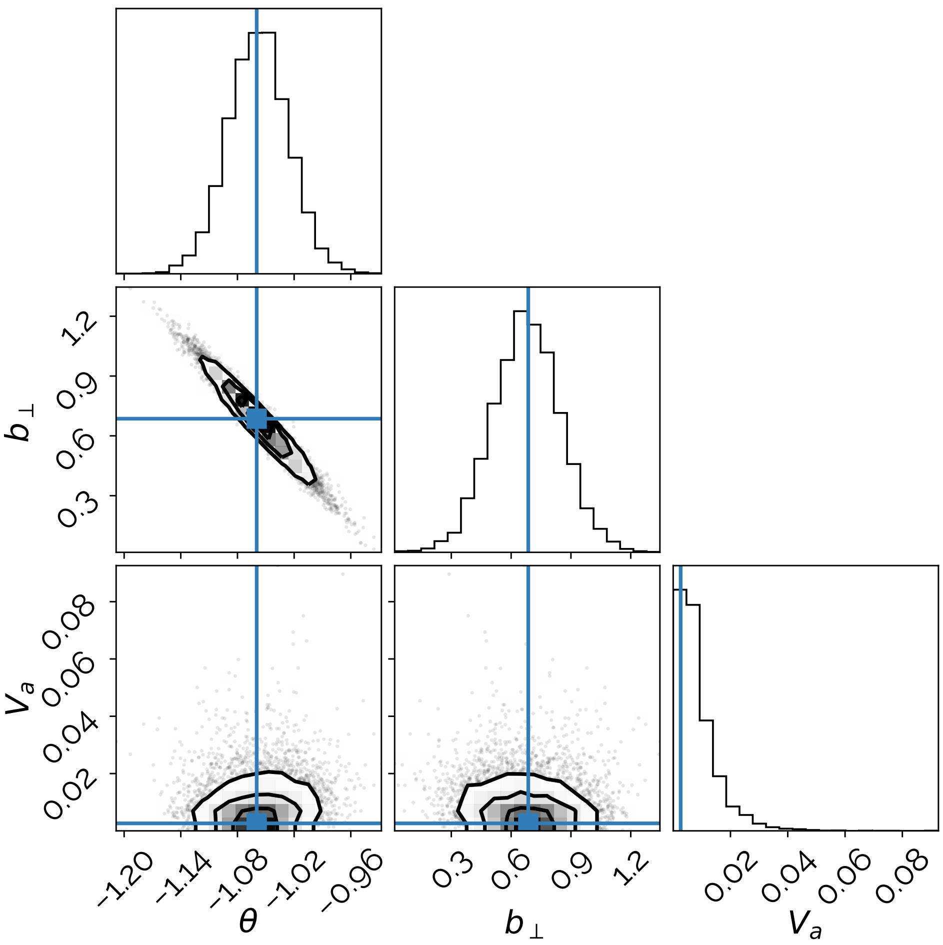

Supplementary Figure 5: **Posterior probability distributions.** For the power law fit to the bedforms on other planetary bodies shown in Fig. 5. The maximum likelihood fit parameters are denoted by the thick, dark blue lines. Note that these are the original fit parameters in orthogonal distance space from the data points (as denoted in Methods), which are later converted to the power law parameters reported in Fig. 4.

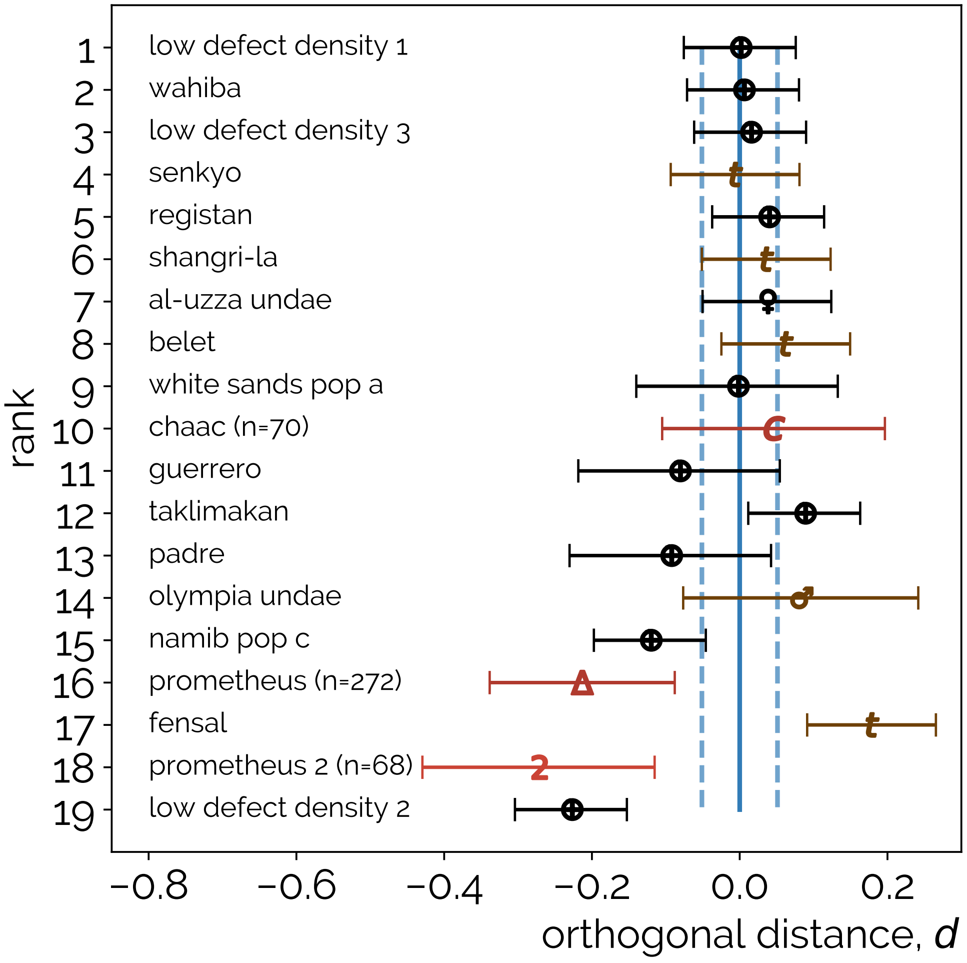

Supplementary Figure 6: Ranked orthogonal distance of crestline defect densities and crest spacing from the power law trend fit to all non-Ionian data. The name of the specific local sites are indicated. The convention for labeling of the data points are the same as that in Fig. 5. All non-Ionian data are from Ewing et al. 2015.[1] The calculation method for the error bars are described in the "Ridge crest digitization" and "Best-fit trendline for defect density to crest spacing ratio" sections of Methods.

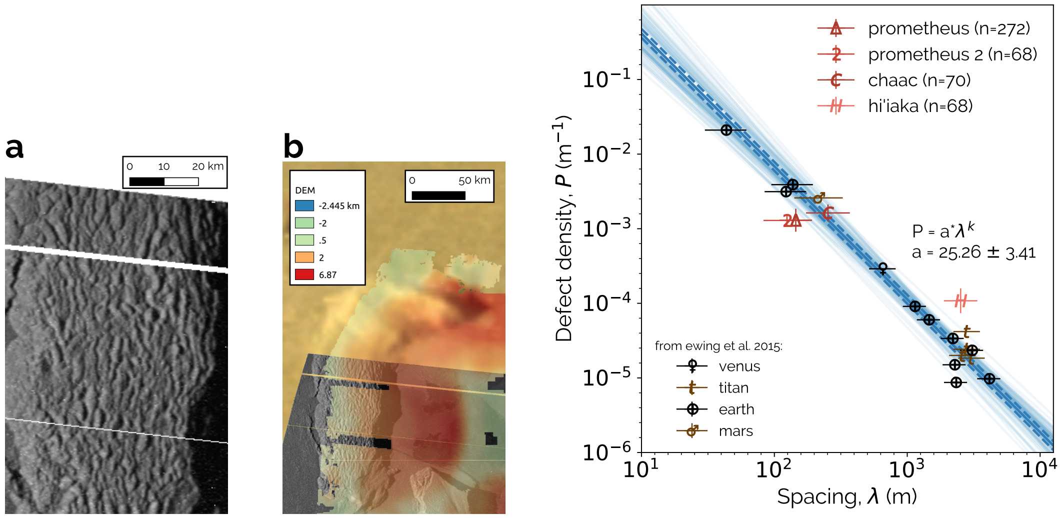

Supplementary Figure 7: **Excluded Hi'iaka Mons ridges.** **a,** *Galileo* Solid State Imager image showing the Hi'iaka ridges. **b** Overlay of the White et al. 2014[2] digital elevation model on the image from **a** at larger scale, showing the ridges occurring on a topographic maximum. **c** The same power law fit to dune bedforms on other planetary bodies shown in Fig. 5, albeit with the Hi'iaka Mons ridges shown for comparison and denoted by the "H".

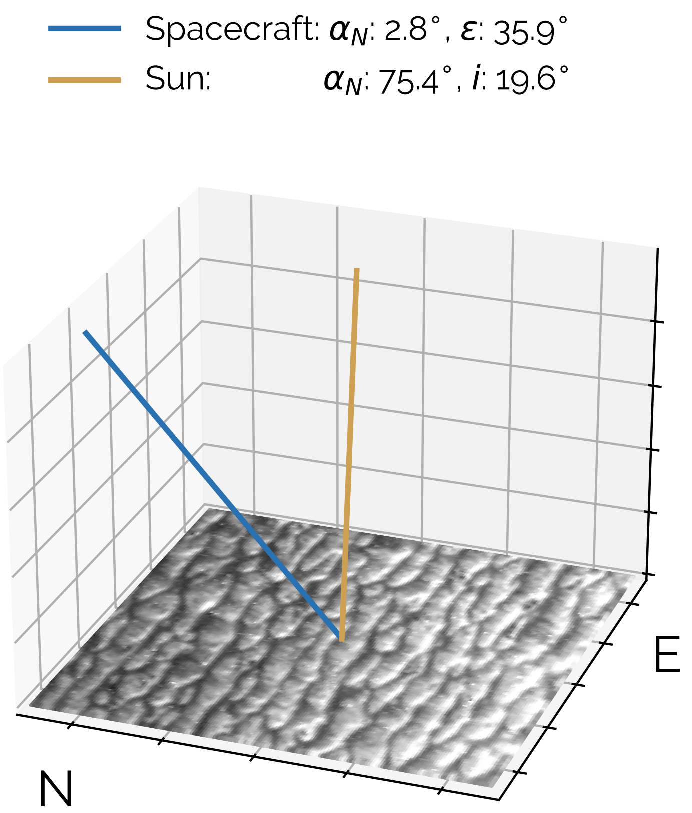

Supplementary Figure 8: **Observation geometry for the Prometheus Patera ridges.** Corresponds to the image in Fig. 3a, and is similar to the geometry for the image in Fig. 3b. The lines trace the rays to the spacecraft and sun. α_N are the north azimuths of the sub-spacecraft and subsolar points on the surface. ϵ is the emission angle of the spacecraft and i is the incidence angle of the sun. Surface cardinal directions are indicated.

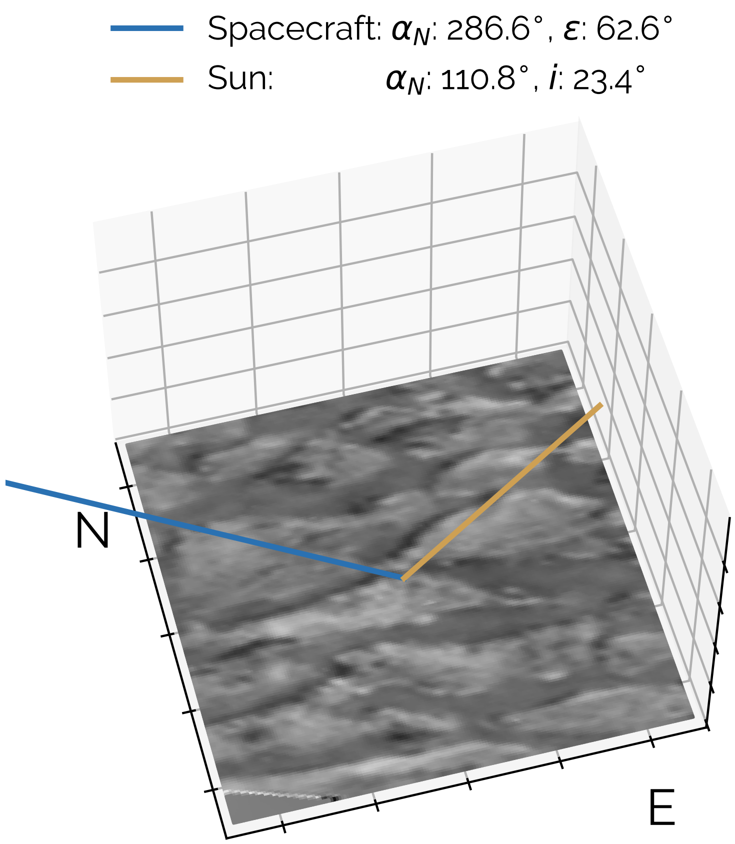

Supplementary Figure 9: Observation geometry for the Chaac Patera ridges. Corresponds to the image in Fig. 3c and Fig. 11. Plotting conventions are the same as in Supplementary Fig. 9.

Supplementary Table 1: Physical constants used in calculations.

Symbol	Parameter	Value	Reference
T_{triple}	Triple point temperature	197.64 K	[3]
P_{triple}	Triple point pressure	1.67×10^3 Pa	[3]
$\rho_{SO2,sol.}$	Density of solid SO_2	1928 kg/m^3	[4]
$\rho_{SO2,liq.}$	Density of liquid SO_2	1620.3 kg/m^3	[3]
ΔH_{fus}	Enthalpy of fusion of SO_2	1.769 cal/mol	[5]
γ_{SO2}	SO_2 specific heat ratio at 288.15 K	1.290	[6]
$\mu_{0,SO2}$	Reference viscosity for SO_2 at 528.57 $^\circ R$	0.01254 cP	[7]
$C_{S,SO2}$	Sutherland's Constant for SO_2	416	[8]

Supplementary Table 2: Data sources used for the ridge sites, as well as calculated parameters. Latitudes and longitudes are the center of the rectangles within which ridges were mapped.The uncertainties in the number of features arise from ambiguity in the linkage of adjacent ridges, see Methods.

Site	Latitude	Longitude	*Galileo* SSI Image ID	Number	Spacing (m)	Defect Density (m^{-1})
Chaac Patera	$12.063°N$	$156.520°W$	C0539932065R	70^{+21}_{-11}	253^{+81}_{-111}	$0.00163^{+0.00043}_{-0.00027}$
Prometheus Patera	$1.935°S$	$154.042°W$	C0539932327R	272^{+93}_{-77}	145^{+42}_{-39}	$0.00129^{+0.00046}_{-0.00034}$
Prometheus Patera 2	$1.953°S$	$153.282°W$	C0539932365R	68^{+13}_{-6}	124^{+69}_{-32}	$0.00129^{+0.00023}_{-0.00015}$
Hi'iaka Ridges	$2.307°S$	$83.354°W$	C0527347478R	68^{+19}_{-7}	2523^{+733}_{-732}	$0.00012^{+0.00004}_{-0.00001}$

Supplementary References

[1] R. C. Ewing, A. G. Hayes, and A. Lucas, "Sand dune patterns on Titan controlled by long-term climate cycles," *Nature Geoscience*, vol. 8, pp. 15–19, Jan. 2015.

[2] O. L. White, P. M. Schenk, F. Nimmo, and T. Hoogenboom, "A new stereo topographic map of Io: Implications for geology from global to local scales: A NEW STEREO TOPOGRAPHIC MAP OF IO," *Journal of Geophysical Research: Planets*, vol. 119, pp. 1276–1301, June 2014.

[3] P. Linstrom and W. Mallard, *NIST Chemistry WebBook,*. No. 69 in NIST Standard Reference Database, Gaithersburg, MD: National Institute of Standards and Technology, 2021.

[4] W. Blitz and O. Huelsmann, "über Molekular- und Atomvolumina. 43. Tieftemperaturdichten kristallisierter Salpetersäure, Schwefelsäure, Phosphorsäure und verwandter Stoffe," *Zeitschrift fuer anorganische und allgemeine Chemie*, 1932.

[5] W. F. Giauque and C. C. Stephenson, "Sulfur Dioxide. The Heat Capacity of Solid and Liquid. Vapor Pressure. Heat of Vaporization. The Entropy Values from Thermal and Molecular Data," *Journal of the American Chemical Society*, vol. 60, pp. 1389–1394, June 1938.

[6] J. G. Speight, *Environmental Analysis and Technology for the Refining Industry.* Hoboken, NJ, USA: John Wiley & Sons, Inc., 2005.

[7] R. C. Weast, *CRC Handbook of Chemistry and Physics.* Boca Raton, FL, USA: Chemical Rubber Company (CRC) Press, Inc., sixty-fifth ed., 1984.

[8] C. Company, "Flow of fluids through valves, fittings, and pipe.," 1988.
